# Dual GIPR and GLP-1R agonist tirzepatide inhibits aeroallergen-induced allergic airway inflammation in mouse model of obese asthma

**DOI:** 10.1111/cea.14252

**Published:** 2022-11-15

**Authors:** Shinji Toki, Jian Zhang, Richard L. Printz, Dawn C. Newcomb, Katherine N. Cahill, Kevin D. Niswender, R. Stokes Peebles

**Affiliations:** 1 Division of Allergy, Pulmonary, and Critical Care Medicine, Vanderbilt University School of Medicine, Nashville, Tennessee, USA; 2 Division of Diabetes, Endocrinology, and Metabolism, Vanderbilt University School of Medicine, Nashville, Tennessee, USA; 3 Department of Pathology, Microbiology, and Immunology, Vanderbilt University School of Medicine, Nashville, Tennessee, USA; 4 Department of Molecular Physiology and Biophysics, Vanderbilt University School of Medicine, Nashville, Tennessee, USA; 5 Tennessee Valley Healthcare System, United States Department of Veterans Affairs, Nashville, Tennessee, USA

**Keywords:** GIP, GLP-1, incretin mimetic drugs, obese asthma, Th2, tirzepatide

## To the Editor,

Nearly 1 in 2 adults is projected to be obese by 2030 in the United States.^1^ Obesity is a risk factor for asthma and is associated with severe, uncontrolled disease. However, there are no pharmacologic therapeutics specifically designated for obese patients with asthma in the current asthma treatment guidelines from the National Heart, Lung, and Blood Institute of the United States National Institutes of Health. In the past two decades, incretin mimetic drugs such as glucagon like peptide-1 receptor (GLP-1R) agonists have been approved for type 2 diabetes. Tirzepatide, a dual gastric inhibitory peptide receptor (GIPR) and GLP-1R agonist, demonstrated greater glycemic control and weight loss than with selective GLP-1R agonists in clinical trials,^2,3^ and was approved as a drug for type 2 diabetes by the U.S. Food and Drug Administration in May 2022.

Our group previously reported that GLP-1R agonist treatment using liraglutide significantly decreased aeroallergen-induced innate allergic inflammation and was associated with a reduction in airway IL-33 release and decreased group 2 innate lymphoid cell (ILC2) activation in lean and obese mouse models.^4,5^ Therefore, we hypothesized that tirzepatide treatment is more effective to decrease allergic inflammation in an obese mouse model compared with either a GLP-1R or GIP single selective receptor agonist treatment. To test this hypothesis, we used a [D-Ala^2^]-GIP (GIPR agonist), semaglutide (GLP-1R agonist), tirzepatide (the dual agonist), or vehicle to treat polygenic obese TALLYHO Jng/J (TALLYHO) mice. Additional information about study methods and findings are available in the following online repository (https://doi.org/10.5281/zenodo.7190225).

**FIGURE 1:**
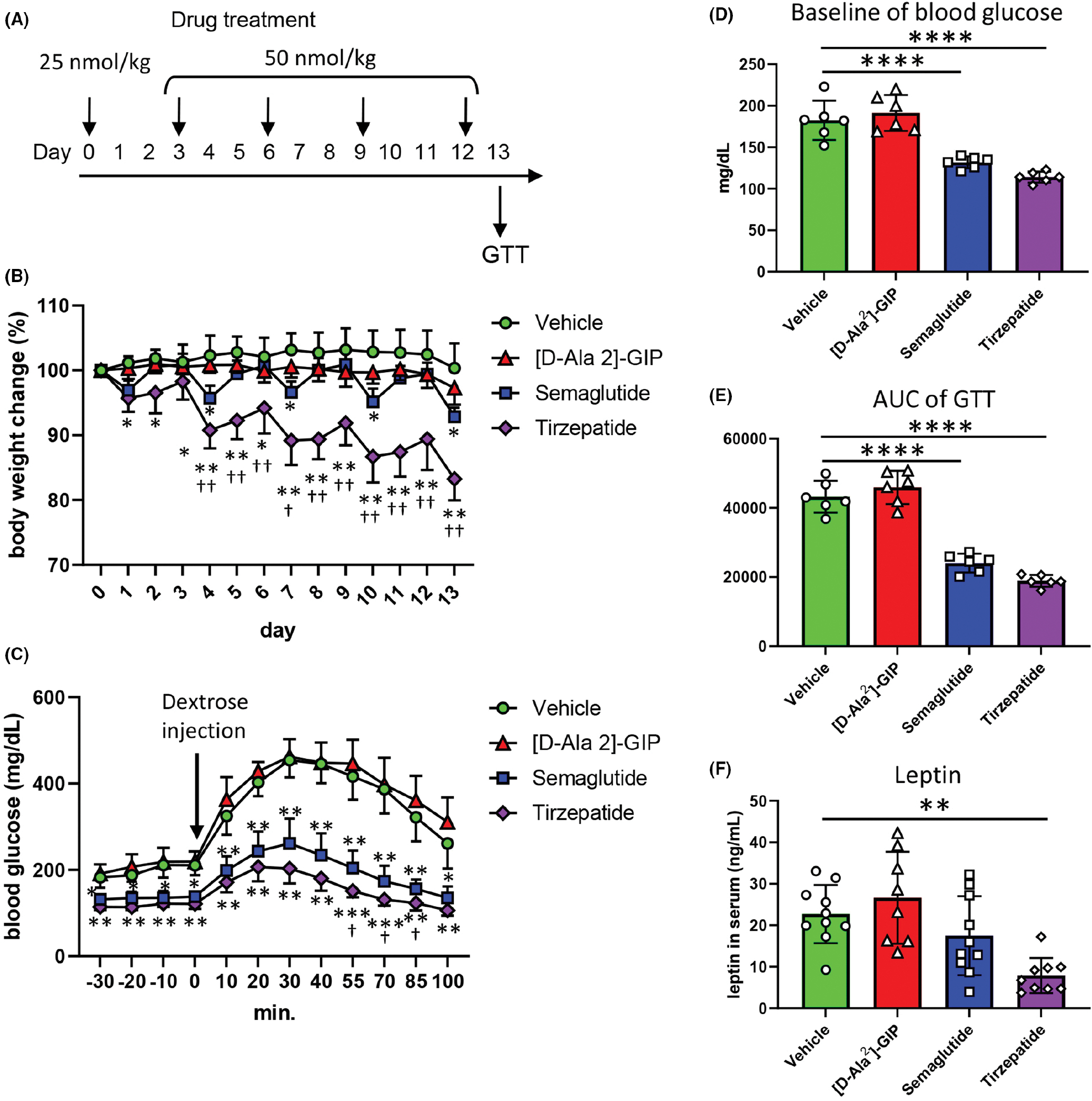
Effects of incretin mimetic drugs treatment for metabolic and glycemic control. (A) Experimental protocol. Incretin mimetic drugs and the vehicle were administered subcutaneously on day 0, 3, 6, 9 and 12. The dose of drugs was 25 nmol/kg on day 0, and 50 nmol/kg on day 3, 6, 9 and 12. (B) The percentage of bodyweight change in response to the incretin mimetic drugs (*n* = 6). (C) Comparison of glucose tolerance after treatment of incretin mimetic drugs (*n* = 6). (D) Blood glucose levels after 6 h fasting (*n* = 6). (E) The area of under the curve (AUC) of glucose tolerance test (GTT) (*n* = 6). (F) Leptin concentrations in serum (*n* = 8–10). The values are the mean ± SD. *p < .05, **p < .01, ***p < .001, ****p < .0001 versus the vehicle. ^†^*p* < .05, ^††^*p* < .01 versus the semaglutide-treated group.

First, we evaluated the effects of the incretin mimetic drugs on metabolic changes in TALLYHO mice. The drug treatment protocol is shown in Figure 1A. [D-Ala^2^]-GIP treatment did not change the bodyweight compared with the vehicle-treated group. Semaglutide treatment temporally decreased the bodyweight 24 h after each treatment, but the weight loss resolved within 48 h after each treatment. In contrast, tirzepatide treatment resulted in a significant decrease of bodyweight compared with the vehicle treatment (Figure 1B). Semaglutide and tirzepatide, but not [D-Ala^2^]-GIP decreased serum glucose levels after fasting for 6 h and glucose tolerance after intraperitoneal injection of dextrose (Figure 1C,D). Further, tirzepatide treatment improved glucose tolerance compared with semaglutide treatment (Figure 1C). The area under the curve (AUC) of a glucose tolerance test (GTT) was significantly decreased by semaglutide and tirzepatide treatment compared with vehicle treatment (Figure 1E). The obesity biomarker, serum leptin, was significantly decreased by tirzepatide treatment, but not the other drug treatment (Figure 1F). Taken together, tirzepatide showed robust improvements in glycemic control and bodyweight in polygenic obese mice.

**FIGURE 2:**
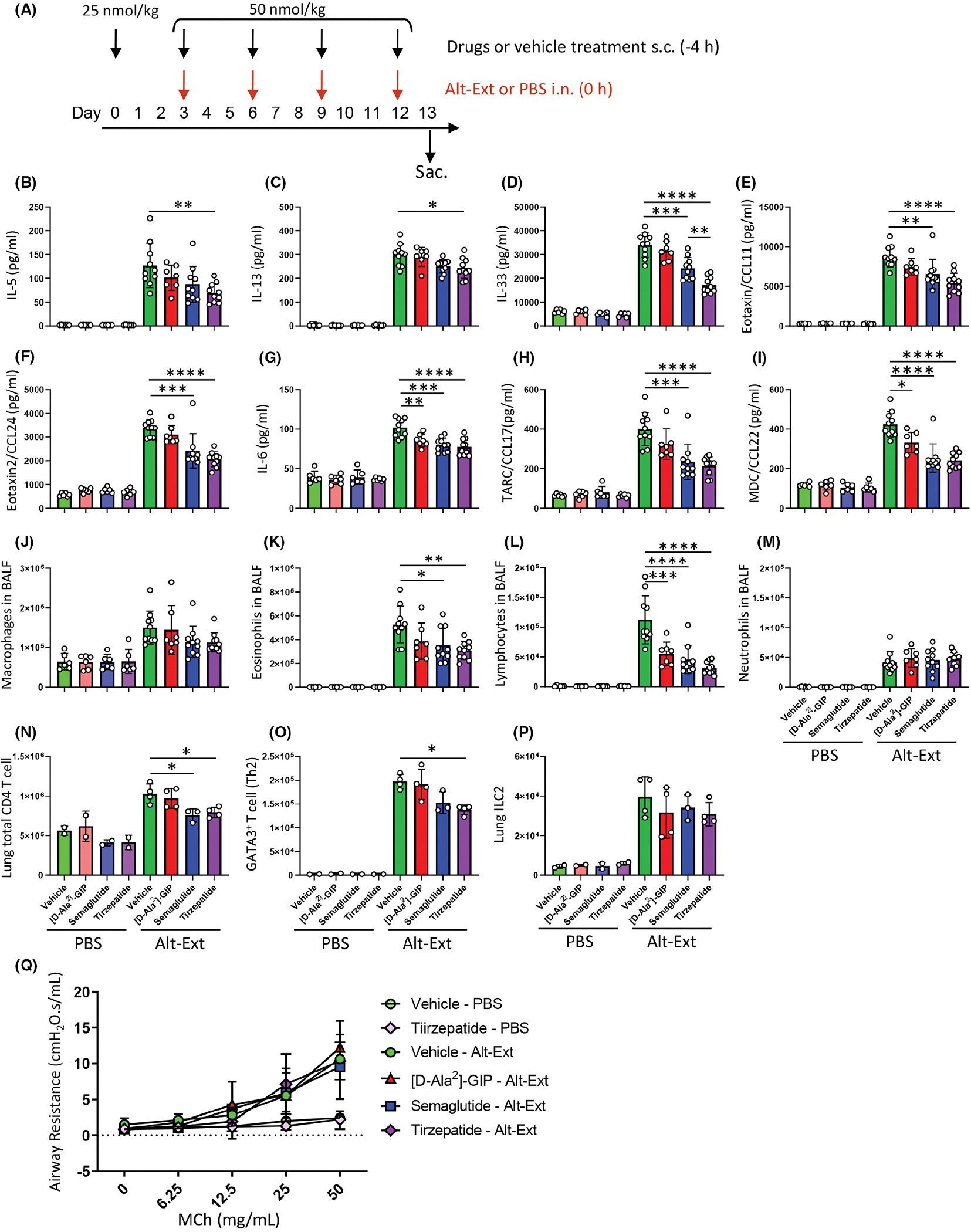
Effects of incretin mimetic drug treatment on Alt-Ext-induced allergic inflammation in the lung. (A) Experimental protocol. The schedule of incretin mimetic drugs treatment is same as Figure 1A. Intranasal challenge of 8 μg (protein amount)/80 μl PBS of Alt-Ext occurred 4 h after the drug treatment on day 3, 6, 9 and 12. The BALF, whole lung, and serum were harvested 24 h after the last Alt-Ext-challenge on day 12. (B–I) Lung homogenates were prepared to measure the protein expression of IL-5, IL-6, IL-13, IL-33, CCL11 (eotaxin), CCL17 (TARC), CCL22 (MDC) and CCL24 (eotaxin-2) by ELISA (*n* = 7–10). (J–M) BALF were harvested 24 h after the last Alt-Ext- or PBS-challenge to evaluate cell differentials, (J) macrophages, (K) eosinophils, (L) lymphocytes and (M) neutrophils. The results shown are the combination of two independent experiments. (N–P) Whole lung cells were prepared to measure the phenotypes of CD4 T cells and ILCs by flowcytometry analysis. (N) Total CD4 T cells, (O) Th2 was identified as GATA3^+^ CD4 T cells, and (P) ILC2. (Q) Airway responsiveness to increasing dose of methacholine challenge. PBS-challenged mice (*n* = 3), Alt-Ext-challenged mice (*n* = 5–6). All results are shown as mean ± SD. *p < .05, **p < .01, ***p < .001, ****p < .0001.

Next, we measured the protein levels of inflammatory cytokines and chemokines in lung homogenates from *Alternaria alternata* extract (Alt-Ext)-or PBS-challenged mice treated with incretin mimetic drugs. The experimental design is shown in Figure 2A. Alt-Ext-challenge significantly increased the protein expression of IL-5, IL-6, IL-13, IL-33, eotaxin (CCL11), eotaxin-2 (CCL24), TARC (CCL17) and MDC (CCL22) in the lung homogenates compared with PBS-challenge (Figure 2B–I). Alt-Ext-induced IL-5 and IL-13 were significantly decreased in the tirzepatide treatment group compared with the vehicle treatment group (Figure 2B,C), but there was no significant difference between tirzepatide and either the [D-Ala^2^]-GIP or semaglutide treatment group. IL-6, IL-33, CCL11, CCL17, CCL22 and CCL24 were significantly decreased in the semaglutide and tirzepatide treatment groups, but not in the [D-Ala^2^]-GIP group, compared with vehicle treatment group (Figure 2D–I). Tirzepatide treatment caused a significant reduction of Alt-Ext-induced IL-33 abundance in lung homogenates (Figure 2D) compared with semaglutide treatment, while there was no statistical difference in the other cytokines and chemokines between the semaglutide and tirzepatide treatment groups. Further, all drug treatments significantly decreased CCL22 and IL-6 compared with vehicle treatment (Figure 2G,I). In contrast, the neutrophil-related chemokines CXCL1 (KC), CXCL5 (LIX), cytokine IL-1β and IL-17 were not decreased in any drug treatment compared with vehicle treatment (See online repository file 1).

To determine the effect of the incretin mimetic drugs treatment for leukocyte infiltration into the airway, we enumerated the cell differentials in bronchoalveolar lavage fluid (BALF) after Alt-Ext- or PBS challenges. Alt-Ext-challenge significantly increased the number of eosinophils, lymphocytes, macrophages and neutrophils compared with PBS-challenged groups (Figure 2J–M). The Alt-Ext-induced eosinophils were significantly decreased in semaglutide and tirzepatide treatment groups compared with vehicle treatment group (Figure 2K), and all drug treatments significantly decreased the number of lymphocytes compared with vehicle treatment (Figure 2L). However, there was no statistical difference in the number of eosinophils and lymphocytes between semaglutide and tirzepatide treatment groups. In contrast, the number of Alt-Ext-induced macrophages and neutrophils were not decreased in any drug treatment (Figure 2J,M).

Our results showed that semaglutide and tirzepatide treatment significantly decreased Alt-Ext-induced Th2-related chemokines CCL17 and CCL22 in the lung (Figure 2H,I), and lymphocyte accumulation into the airway compared with vehicle treatment (Figure 2L). To confirm whether the incretin mimetic drugs down-regulate CD4 T cell development or control the phenotype of CD4 T cell and innate lymphoid cells (ILCs), we measured the number of lung Th2, T reg, ILC2 and ILC3 from mice subjected to the same experimental protocol as depicted in Figure 2A. The gating strategies of total CD4 T cells, GATA3^+^ CD4 T cells (Th2), and Foxp3^+^ CD4 T cells (T reg), ILC2, and ILC3 are shown in online repository files 2 and 3. Alt-Ext-challenge significantly increased the number of total CD4 T cells, Th2, Treg and ILC2, but not ILC3 compared with PBS-challenge (Figure 2N–P, online repository file 4). Semaglutide and tirzepatide treatment significantly decreased the number of total CD4 T cells in the lung compared with vehicle treatment group (Figure 2N). Further, tirzepatide treatment significantly decreased the number of Th2 in the lung, but neither [D-Ala^2^]-GIP nor semaglutide treatment showed a decrease of Th2 compared with vehicle treatment. In contrast, no drug treatment affected the number of Treg, ILC2 and ILC3 in the lungs in Alt-Ext-challenged mice (Figure 2P, online repository file 4). Further, we tested whether incretin mimetic drugs treatment decreased airway responsiveness (AR) in Alt-Ext- or PBS-challenged TALLYHO mice. Alt-Ext-challenge significantly increased methacholine-induced AR compared with PBS-challenge. Meanwhile, there was no significant differences in Alt-Ext-induced AR response to methacholine between any incretin mimetic drugs and the vehicle treatment groups (Figure 2Q).

To determine the effect of incretin mimetic drugs treatment as a therapeutic intervention, the drug treatment was started on the same day of 2nd Alt-Ext-challenge (day 3) (See online repository file 5A). In this model, Alt-Ext-induced lung IL-6, IL-13, IL-33, CCL22, and the number of lymphocytes in the BALF were significantly decreased in semaglutide and tirzepatide treatment groups compared with vehicle treatment group. In addition, CCL11 in the lung homogenates and the number of eosinophils in BALF were significantly decreased in tirzepatide treatment group compared with vehicle treatment group. [D-Ala^2^]-GIP treatment did not decrease any cytokines in the lung and the number of cells in BALF (See online repository file 5B–M). Taken together, tirzepatide treatment significantly decreased Alt-Ext-induced type 2 inflammation mediated by reduction of Th2 development in the lung compared with vehicle treatment.

At first, we hypothesized that GLP-1R and GIPR dual agonist treatment is more effective in reducing aeroallergen-induced allergic lung inflammation compared with either selective GLP-1R or GIPR agonist treatment. Our results showed that GIPR agonist [D-Ala^2^]-GIP treatment did not affect bodyweight and eosinophilia in the airway. Although the half-life of semaglutide and tirzepatide is approximately 165 and 116 h, the peak of [D-Ala^2^]-GIP in the plasma was reported to be 1 h, but disappeared at 24 h, after the subcutaneous injection.^6^ Since [D-Ala^2^]-GIP likely has a shorter half-life than semaglutide and tirzepatide, the treatment schedule in our study might be inappropriate for [D-Ala^2^]-GIP to affect bodyweight, glucose tolerance, and Alt-Ext-induced type 2 inflammation.

Treatment of semaglutide and tirzepatide significantly decreased Alt-Ext-induced expression of CCL11, CCL17, CCL22, CCL24, IL-6 and IL-33 in the lung, the number of eosinophils and lymphocytes in the BALF, and CD4 T cells in the lung compared with vehicle treatment. However, there was no statistical differences in these type 2 inflammatory markers between semaglutide and tirzepatide treatment except for IL-33. Dual agonist tirzepatide treatment resulted in a limited-benefit of anti-allergic inflammatory effects compared with selective GLP-1R agonist semaglutide treatment. Although tirzepatide can bind both GLP-1R and GIPR, the affinity level is not equal to the endogenous ligand GLP-1 and GIP. Previous studies reported that tirzepatide had less affinity to GLP-1R compared with different selective GLP-1R agonists in an in vitro assay.^7^ The GLP-1R binding affinity of tirzepatide might be less than the affinity of semaglutide in our in vivo mouse model. Meanwhile, our results showed that Alt-Ext-induced IL-5, IL-13 and the number of lung Th2 were statistically decreased in the tirzepatide treatment group, but not in the other drugs treatment groups compared with vehicle treatment group. These results indicate a potential advantage of tirzepatide for decreasing allergic inflammation through the reduction of Th2 accumulation in the lung.

Previous studies showed that over 10% bodyweight loss improved Asthma Control Questionnaire (ACQ) score in obese patients with severe asthma.^8^ Therefore, non-pharmacologic therapies to reduce the bodyweight are recommended for obese asthma patients in current asthma treatment guidelines. Data from phase 3 studies of tirzepatide supports that weight loss in excess of 20% can be achieved.^3^ Therefore, tirzepatide-induced bodyweight reduction might improve asthma control in obese asthma patients. In our model, there was no differences in the physical activity of the groups of mice between any of the incretin drug treated and the vehicle treatment groups. To confirm whether the anti-tirzepatide-specific antibodies are developed and have an influence for physical activity, we measured anti-tirzepatide-specific IgG1 in the serum from mice treated with tirzepatide or the vehicle. Although Alt-Ext-challenged mice had a greater level of anti-Alt-Ext-specific IgG1 compared with PBS-challenged mice, tirzepatide-treated mice had no increase of anti-tirzepatide-specific IgG1 compared with vehicle treated mice (See online repository file 6). This result showed that tirzepatide treatment did not induce the drug-specific adaptive immune responses.

Our results and human clinical study data confirmed that tirzepatide treatment decreased serum leptin level as well as bodyweight without adversely affecting physical activity. Since leptin increases the activation of Th2 and ILC2 to enhance type-2 inflammation,^9^ the reduction of leptin by tirzepatide treatment might be one mechanism to reduce the activation of Th2 cells to suppress allergic lung inflammation.

Our findings revealed that GLP-1R and GIPR dual agonist, tirzepatide, had anti-allergic inflammatory effects for the aeroallergen-induced model in obese asthma. These findings indicate that incretin mimetic drugs, especially tirzepatide might be a new therapeutic strategy for obese patients who have asthma.

## Data Availability

The data that support the findings of this study are openly available in zenodo at https://doi.org/10.5281/zenodo.7190225.

## Key Message

- The dual GIPR and GLP-1R agonist tirzepatide decreased bodyweight and hyperglycemia in polygenic obese mice.
- Tirzepatide down-regulated aeroallergen-induced type 2 inflammation.
- Tirzepatide may be a potential therapeutic strategy for obese patients with asthma.
